# High-Throughput Identification and Analysis of Novel Conotoxins from Three Vermivorous Cone Snails by Transcriptome Sequencing

**DOI:** 10.3390/md17030193

**Published:** 2019-03-26

**Authors:** Ge Yao, Chao Peng, Yabing Zhu, Chongxu Fan, Hui Jiang, Jisheng Chen, Ying Cao, Qiong Shi

**Affiliations:** 1 State Key Laboratory of NBC Protection for Civilian, Beijing 102205, China; bzyaoge@163.com (G.Y.); chongxu_fan@hotmail.com (C.F.); jiangtide@sina.cn (H.J.); chenjsh@cae.cn (J.C.); 2 Shenzhen Key Lab of Marine Genomics, Guangdong Provincial Key Lab of Molecular Breeding in Marine Economic Animals, BGI Academy of Marine Sciences, BGI Marine, BGI, Shenzhen 518083, China; pengchao@genomics.cn; 3 BGI Genomics, BGI-Shenzhen, Shenzhen 518083, China; zhuyabing@genomics.cn; 4 Laboratory of Aquatic Genomics, College of Life Sciences and Oceanography, Shenzhen University, Shenzhen 518060, China

**Keywords:** *Conus*, conotoxin, transcriptome sequencing, phylogeny, venom duct

## Abstract

The venom of each *Conus* species consists of a diverse array of neurophysiologically active peptides, which are mostly unique to the examined species. In this study, we performed high-throughput transcriptome sequencing to extract and analyze putative conotoxin transcripts from the venom ducts of 3 vermivorous cone snails (*C. caracteristicus*, *C. generalis*, and *C. quercinus*), which are resident in offshore waters of the South China Sea. In total, 118, 61, and 48 putative conotoxins (across 22 superfamilies) were identified from the 3 *Conus* species, respectively; most of them are novel, and some possess new cysteine patterns. Interestingly, a series of 45 unassigned conotoxins presented with a new framework of C-C-C-C-C-C, and their mature regions were sufficiently distinct from any other known conotoxins, most likely representing a new superfamily. O- and M-superfamily conotoxins were the most abundant in transcript number and transcription level, suggesting their critical roles in the venom functions of these vermivorous cone snails. In addition, we identified numerous functional proteins with potential involvement in the biosynthesis, modification, and delivery process of conotoxins, which may shed light on the fundamental mechanisms for the generation of these important conotoxins within the venom duct of cone snails.

## 1. Introduction

Cone snail is the common name for predatory marine mollusks in the family Conidae, with over 700 extant species and a categorization of four genera and 71 subgenera [1,2,3]. Within the *Conus*, the largest genus in the Conidae, 57 subgenera have been recognized [3]. As venomous predators distributed throughout tropical and subtropical coastal waters all over the world, the living cone snails are typically divided into 3 groups based on their feeding habits, including fish hunters, mollusc hunters, and worm hunters [4,5,6]. Some phylogenetic data have suggested that the ancestral cone snails preyed on marine worms [7,8]. The fish-hunting and mollusc-hunting groups account for ~30% of *Conus* species, and they are assumed to be dangerous to humans; however, the largest worm-hunting group seems to be nonthreatening [5,9,10]. An analysis of 141 human injuries reported from 34 responsible *Conus* species during the period of 1670–2017 [11] supports the fact that the venom of worm-hunting cone snails has only mild effects on humans, compared with those from fish-hunting and mollusc-hunting groups.

Although they are slow-moving creatures, cone snails can defeat fast-moving preys, competitors, and predators because of their specialized envenomation apparatus with potent venom components [12,13,14]. These venom components are commonly named conotoxins, a unique and remarkably diverse group of bioactive peptides with various pharmacological functions [6,14,15,16,17], which target a wide variety of ion channels, receptors, and even their subtypes in preys, predators, and humans with high affinity and specificity [6,18,19,20]. Consequently, conotoxins have become a research hotspot for the treatment of various neuropathic diseases, such as neuralgia, epilepsy, addiction, and Parkinson’s disease [6,21,22,23,24,25,26,27,28,29,30,31].

With popular estimates of 50~200 classical conotoxins in a single *Conus* species, more than 80,000 natural conotoxins may exist in cone snails on a global scale [32,33,34]. Recent studies have shown that new methods, such as mass spectrometry, next-generation sequencing (NGS), and bioinformatics technologies, have predicted hundreds to thousands of venom peptides or transcripts from a single *Conus* species [34,35,36,37]. Therefore, cone snails, a tremendous store of natural conotoxins, are an underexploited resource for the development of potential drug candidates to treat a wide variety of human diseases [38].

Our present work reports a high-throughput transcriptome research on 3 worm hunting *Conus* species, *C. (Puncticulis) caracteristicus* (*C. caracteristicus*), *C. (Lividoconus) quercinus* (*C. quercinus*) and *C. (Strategoconus) generalis* (*C. generalis*), which are resident in offshore waters of the South China Sea. To date, there have been few studies on the screening of conotoxins from cone snails by transcriptome sequencing. One of our earlier studies on *C. quercinus* identified 65, 52, and 55 conotoxins from the venom duct, venom bulb and salivary gland, respectively [39]. Furthermore, only 38 and 4 conotoxins have been previously identified in *C. caracteristicus* and *C. generalis*, with classification into 10 (A, I3, M, O1, O2, O3, Q, S, T and Y) and 2 (D, O1) superfamilies, respectively [40,41,42,43,44,45,46]. In order to improve our understanding of the diversity of conotoxins, we performed transcriptome sequencing for the high-throughput identification and analysis of conotoxins from the venom duct of the 3 frequently collected cone snails.

## 2. Results

### 2.1. Summary of De Novo Assembled Transcriptome Data

After removal of low-quality reads, ambiguous reads and adapter sequences, we generated 4.57, 3.21, and 4.37 gigabases (Gb) of clean reads (with a mean length of 90 bp) for the venom duct transcriptomes of the 3 *Conus* species. Corresponding quality score 20 (Q20) of these sequencing data were 95.99%, 98.31%, and 96.00% respectively (Table 1). *De novo* assembling of all the high-quality clean reads using SOAPdenovo produced 213 k, 153 k, and 219 k contigs for the 3 species, respectively, which were subsequently assembled into scaffolds and unigenes. In total, the assembly of each transcriptome possessed 72 k, 61 k, and 95 k unigenes. More details of scaffold number, unigene number, mean length, and N50 value are summarized in Table 2.

### 2.2. Screening of Conotoxins in the Venom Duct Transcriptomes

To annotate conotoxin coding sequences among the unigenes, we searched all six-frame translations of the unigenes against a local reference database of known conotoxins constructed from the public ConoServer database by running Genewise and Agustus with an E-value cut-off of 1.0 × 10^−5^ [18], and then manually checked them using the ConoPrec tool [19]. After the removal of the transcripts with duplication, frame-shifting, and truncated mature region sequences, we identified 118, 61, and 48 putative conotoxin sequences from the 3 transcriptome datasets of *C. caracteristicus*, *C. generalis,* and *C. quercinus*, respectively (Table 3, Table 4 and Table 5). Interestingly, most of these sequences are reported for the first time and some possess new cysteine patterns. We then summarized and named these predicted conotoxins from the 3 *Conus* species as Ca-1 to Ca-118, Ge-1 to Ge-61, and Qu-1 to Qu-48, respectively (see more details in Supplementary Tables S1–S3).

**Figure 1:**
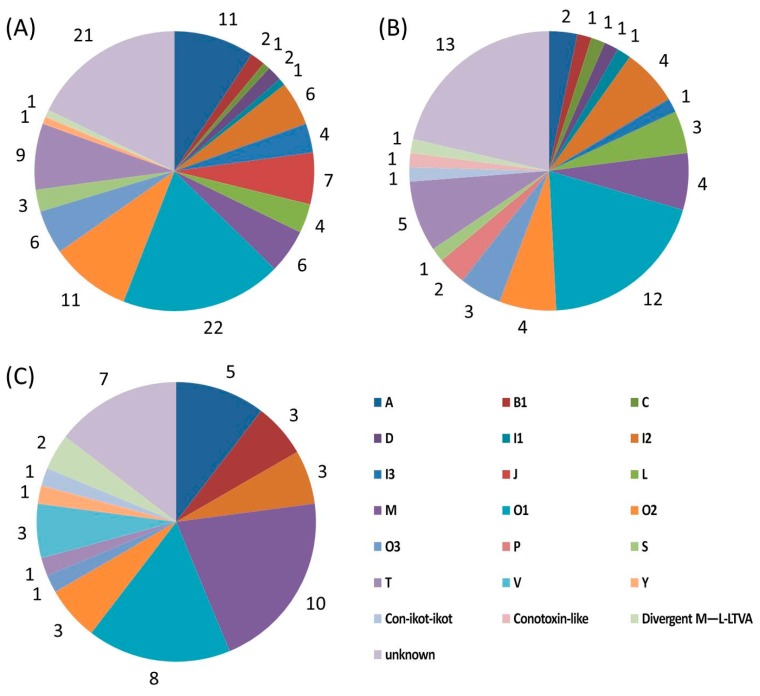
Summary of the conotoxins identified from the 3 *Conus* species. Many superfamilies or groups of conotoxins were classified in (**A**) *C. caracteristicus*, (**B**) *C. generalis*, and (**C**) *C. quercinus*.

In this study, each putative conotoxin was assigned to a superfamily based on its percentage of sequence identity to the highly conserved signal region of the known superfamily from the public ConoServer database (Figure 1). Here, among the 118 putative conotoxins in *C. caracteristicus*, 96 sequences were assigned to 16 previously reported superfamilies (A, B1, C, D, I1, I2, I3, J, L, M, O1, O2, O3, S, T, and Y), while only 1 sequence was classified into the “divergent M—L-LTVA” superfamily. In addition, 21 sequences were not assigned to any known superfamily (named “unknown”; see more details in Table 3).

Among the 61 putative conotoxins in *C. generalis*, 46 sequences were classified into 15 known superfamilies (A, B1, C, D, I1, I2, I3, L, M, O1, O2, O3, P, S, and T) and 1 cysteine-rich con-ikot-ikot family. In addition, 1 sequence was assigned to the “divergent MSTLGMTLL-” super-family, 1 was assigned to the conotoxin-like group, and the other 13 sequences were unknown (see more details in Table 4).

Compared with the conotoxin sequences reported in our previous transcriptome study of *C. quercinus* [39], the number of conotoxins from *C. quercinus* in this study was less, with the identification of only 48 putative conotoxins. Among them, 39 sequences were classified into 10 known superfamilies (A, B1, I2, M, O1, O2, O3, T, V, and Y) and the con-ikot-ikot family, 2 sequences were assigned to the “divergent M—L-LTVA” superfamily, and the remaining 7 sequences were unknown (see more details in Table 5).

### 2.3. Quantification of Conotoxin Abundance

**Figure 2:**
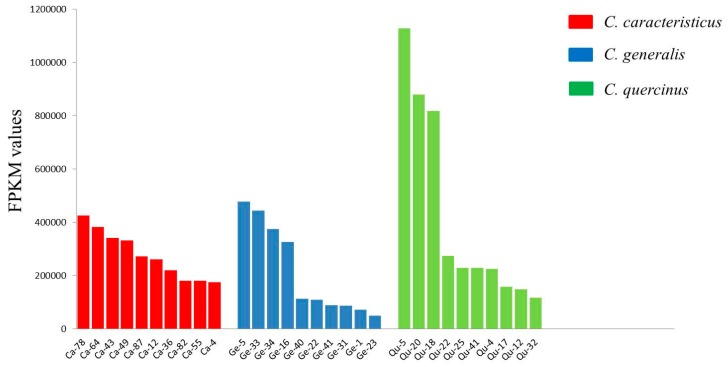
Comparison of the top 10 conotoxins (with the highest FPKM values) from the 3 transcriptome datasets.

To investigate the transcription levels of conotoxins in each species, we mapped clean reads back to the *de novo* assembled unigenes and calculated the fragments per kilobase of transcript per million mapped fragments (FPKM) values to quantify the abundance of each conotoxin transcript. We screened out those conotoxins with high transcription abundance, and the top 10 (with the highest FPKM values) were selected from each dataset for comparison. The number of mapped reads for the top 10 conotoxins accounted for 60.6%, 84.9%, and 80.4% of the total conotoxin reads from *C. caracteristicus*, *C. generalis*, and *C. quercinus* respectively. Interestingly, O- and M-superfamilies were always the most abundant within each transcriptome dataset (Figure 2, Supplementary Table S4), suggesting their critical roles in predation and defense for the 3 vermivorous *Conus* species.

**Figure 3:**
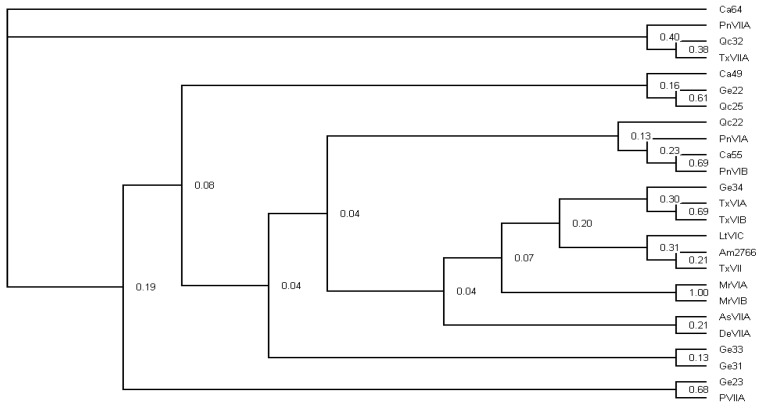
A Bayesian phylogenetic tree of the O-superfamily conotoxins. Posterior probabilities are labeled at each node.

The O-superfamily conotoxins specifically target a wide range of ion channels and receptors. In this study, a Bayesian phylogenetic tree was constructed with the Markov Chain Monte Carlo algorithm to analyze the relationships among these predicted O-superfamily conotoxins (Figure 3), in which 14 were presented with known bioactivities (previously reported from different *Conus* species) and 11 demonstrated high transcription levels from this study. Our phylogenetic assessment indicated that the O-superfamily conotoxin clades from the same *Conus* species arise as distinct lineages, suggesting that there was no correlation between the evolution of conotoxin sequences and interspecific genetic relationship (see more details in Figure 3). In turn, Ca-55 and ω-conotoxin PnVIA/PnVIB formed a monophyletic clade [47], and the Posterior probability of Ca-55 and PnVIB was 0.69. Ge-23 and κ-conotoxin PVIIA formed an individual clade [48], and the Posterior probability of both was 0.68. Qc-32 and γ-conotoxin PnVIIA/TxVIIA formed a monophyletic clade [49,50], but the homology between them was not supported by the low Posterior probability; they all exhibited distant evolutionary relationships to the other O-super-family conotoxins. Similarly, Ge-34 and δ-conotoxin TxVIA/TxVIB also formed a separate clade but with low Posterior probability [51].

### 2.4. Diversity of Conotoxin Structures

Among the putative conotoxin sequences in the 3 venom duct transcriptomes, most of them were discovered for the first time, and some possessed new cysteine frameworks or belonged to unknown superfamilies. The O-superfamily, including O1, O2, and O3, was the most abundant group in terms of conotoxin number (Table 3, Table 4 and Table 5). All of the O1- and O3-superfamily members exhibited the conventional VI/VII (C-C-CC-C-C) cysteine framework, which provides a stable three-disulfide inhibitor cysteine knot (ICK) motif [46]. Four O2-superfamily sequences from *C. caracteristicus* and *C. generalis* exhibited a C-terminal elongated XV (C-C-CC-C-C-C-C) cysteine framework (see Table 3 and Table 4), and 5 short single disulfide-containing contryphan peptides with high identity were identified from only *C. caracteristicus* (Table 3). Interestingly, 5 O-superfamily members had the same mature regions as reported sequences from other *Conus* species. For example, Ge-24 from *C. generalis* had exactly the same mature peptide and prepro-region as the reported MgJr94 from piscivorous *C. magus* [52], while Ge-22 and Ge-23 showed mutations in the pro-peptide regions but with identical mature peptides to MiK41 and MiK42 respectively from *C. miles* [53].

The M-superfamily was also the predominant one in terms of transcription abundance and diversity of cysteine frameworks. Besides 17 sequences with the typical III (CC–C–C–CC) cysteine pattern, 3 conotoxins with IV (CC-C-C-C-C), XVI (C-C-CC) and XXVII (C-CC-C-C-C) cysteine frameworks were also observed (Table 3, Table 4 and Table 5).

For the I-superfamily, a variety of conotoxin (including I1, I2 and I3) transcripts were retrieved from the 3 transcriptomes. A total of 20 members were identified, in which most (13 sequences) belonged to the I2-superfamily and possessed the typical post-peptide and pro-region-free structure. These I-superfamily conotoxins generally had various signal regions and cysteine-rich frameworks, of which 11 exhibited the representative XI (C-C-CC-CC-C-C) pattern and 7 possessed the XII (C-C-C-C-CC-C-C) pattern, whereas only 2 had the framework VI/VII (C-C-CC-C-C) with distinct signal sequences and loop length (Table 3, Table 4 and Table 5).

Concerning the A-superfamily, 18 conotoxins were determined with 15 sequences of the common I (CC-C-C) pattern, and 3 transcripts with only 3 cysteine residues (the CC-C framework). In contrast to notable abundance and variety of A-superfamily conotoxins in previously reported piscivorous species [54,55,56], the number and diversity of the A-superfamily identified from these 3 vermivorous species were scarce. Meanwhile, 15 T-superfamily conotoxins were also identified; however, most of them exhibited the simple V (CC-CC) framework with high identity to several known τ-conotoxins, and only one contained the XVI (C-C-CC) framework.

In addition to the abovementioned major superfamilies, many less representative B1 (conontokin)-, C (contulakin)-, D-, J-, L-, P-, S-, V-, Y-superfamilies and the con-ikot-ikot family were also discovered in the 3 transcriptomes. For example, 6 conontokin sequences and 2 contulakin sequences with the cysteine free pattern were identified in this study. Three D-superfamily sequences with the XII (C-CC-C-CC-C-C-C-C) and XV (C-C-CC-C-C-C-C) patterns, 3 V-superfamily sequences with the XV (C-C-CC-C-C-C-C) pattern, and 2 Y-superfamily sequences with the XVII (C-C-CC-C-CC-C) pattern were also observed; interestingly, these 3 superfamilies have been isolated only from vermivorous cone snails to date [57]. Meanwhile, 2 con-ikot-ikot peptides with the novel CC-C-C-C-CC-C-C-C and CC-C-C-C-C-CC-C-C-C-C frameworks were identified. Con-ikot-ikot toxins were reported to specifically target post-synaptic AMPA receptors [58], but in consideration of the complex cysteine patterns and variable loop length, the functions of both conotoxins with new cysteine frameworks are worth investigating further.

**Figure 4:**
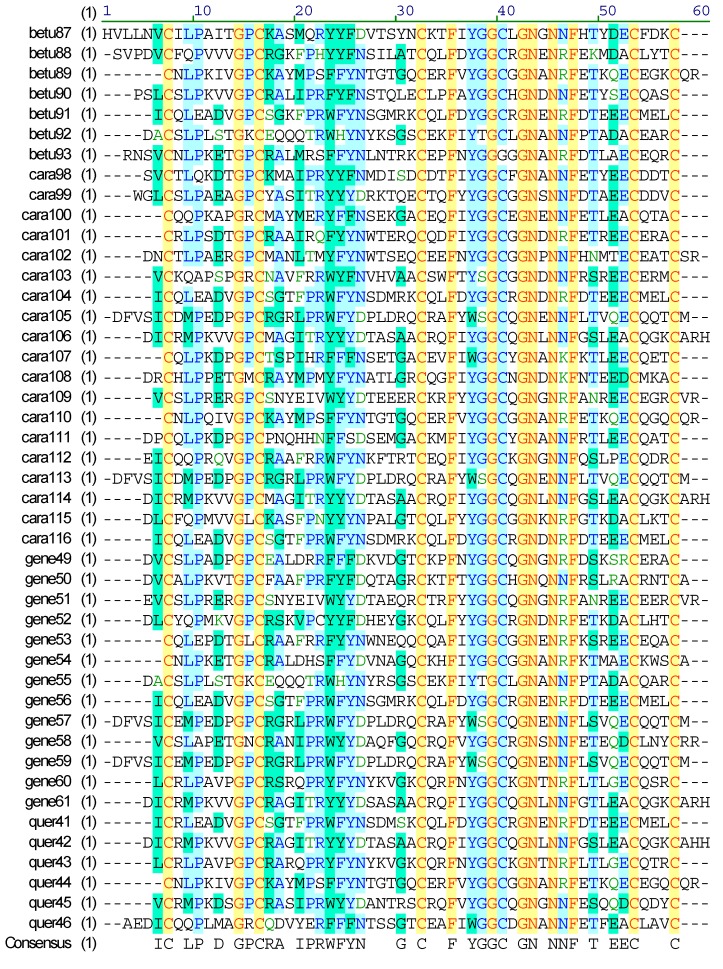
Alignment of the achieved new superfamily conotoxins from venom duct transcriptomes of *C. betulinus* [32] (betu), *C. caracteristicus* (cara), *C. generalis* (gene), and *C. quercinus* (quer).

In this study, combined with our published report of *C. betulinus* [32], we also identified 45 putative unassigned conotoxins, which possessed the IX (C-C-C-C-C-C) cysteine framework and loop lengths same as Cal9.1a~d from *Californiconus californicus* [3,59]. In fact, Cal9.1a~d contained unassigned signal peptide sequences, and their loop lengths and mature regions appeared to be sufficiently distinct from other known conotoxins. This group of conotoxins was previously found only in *Californiconus californicus*, an unusual species with special prey-capture behavior and prey preferences, and phylogenetic analysis also indicated that *Californiconus californicus* has large evolutionary distance from the *Conus* species [60]. The experts at WoRMS placed this genus *Californiconus* in the family Conidae, but as indicated it is highly divergent from the Conidae; hence, some researchers have placed this genus in a proposed separate (sub)family [1]. Our present work confirmed that these types of conotoxins are possibly synthesized in various species with different feeding habits; therefore, they may represent a new superfamily with potential specificity in pharmacological activity (Figure 4).

### 2.5. Identification of Conotoxin Biosynthesis Related Proteins

Transcripts for genes encoding functional proteins that are potentially involved in conotoxin biosynthesis were also annotated in the 3 transcriptome datasets. By homology comparison to known post-translational modification enzymes, we presumed that multiple proteins possess enzymatic activities for involvement in conotoxin maturation and modification in the venom duct lumen. From the transcriptomes, we also identified some isoforms of endoprotease, including sequences with high similarity to Tex31 [61], which has the hydrolytic activity to separate mature conotoxins from the precursor constituents.

Formation of disulfides is the most ubiquitous modification within conotoxins. This process and related proper peptide-folding are mediated by protein disulfide isomerases (PDIs), peptidyl- prolyl cis-trans isomerases, immunoglobulin-binding proteins, and chaperones (e.g., hsp70, hsp60, and calreticulin) [33]. All of these proteins, especially multi-isoforms of PDI were identified (Supplementary Tables S1–S3). Complete sequences of peptidylglycine alpha-amidating monooxygenase with two domains were identified as well, which may mediate the C-terminal amidation process for the full activity of various neuroactive peptides [62]. In addition, other candidate enzymes participating in post- translational modification were also predicted, such as prolyl/lysyl-hydroxylase, vitamin K-dependent γ-carboxylase, and Glutaminyl-peptide cyclotransferase.

Our data also revealed numerous sequences with potential roles in transportation, synergy, and degradation of conotoxins. Translocator-like sequences including the Sec family and diverse transmembrane proteins were discovered. In particular, we predicted several transcripts with high similarity to the Sec61 and Sec14 translocon that were identified in the spider venom duct with the ability to bind specific polypeptide toxins and to induce subsequent localization and transportation [63,64]. Large arrays of conotoxin-related proteins (widely existing in other animal venoms) with high transcription levels were also predicted. These sequences include multiple enzymes, such as the phospholipase A2 (PLA2) family, nucleotidase and hyaluronidase, as most of them may have neurotoxic and cytotoxic activities themselves or participate in anti-hemostatic effects [65], and may enhance the diffusion of conotoxins and cooperate with them for prey capture or synergistic predator defense. Meanwhile, many proteins homologous to the ubiquitin and ubiquitylation system were retrieved from the 3 transcriptome datasets in this study. These proteins may contribute to the degradation of poor-quality conotoxin molecules synthesized in the venom duct, which will ensure the effectiveness of venom to a higher degree in the prey capture process [66] (Supplementary Table S5).

## 3. Discussion

A total of 118, 61, and 48 putative conotoxin transcripts were identified from the 3 transcriptome datasets of *C. caracteristicus*, *C. generalis,* and *C. quercinus*, respectively. Given that these *Conus* species have similar feeding habits and distribution (in the offshore waters of the South China Sea), the interspecific divergence in toxin numbers and transcription levels were beyond our expectation. The interspecies variability of venom may contribute to the dietary preferences for different worms. Furthermore, the conotoxin composition in the venom duct of *C. quercinus* from this study was only 23% (11 of 48), identical to our previous report [39]. This remarkable variance of conotoxin types in the same species, especially in the same organ and using the same sequencing method, may be due to differences between *Conus* individuals that were from different geographical populations or at different developmental stages (for a more detailed discussion, please refer to our previous report on *C. betulinus* [32]). The intraspecies variability of venom composition has also been recently observed by other researchers, who recommended sequencing transcriptomes of more than one individual for the solid analysis of the conotoxin inventory in any examined species [67]. In addition, we also identified some identical conotoxin sequences from different *Conus* species; this convergent evolution trend is widespread presented among various *Conus* species with diverse phylogenetic clades. Related biological significance needs more investigation.

Conotoxins have a variety of mechanisms for actions, which however have been limited by the lack of high-throughput functional screening methods; therefore, most of them have not been determined by far. In this study, we attempted to apply the phylogenetic analysis strategy to explore the evolutional relationships of these highly transcribed O-superfamily members, and then predicted their potential bioactivities. Among the top 10 conotoxins with the highest FPKM values in the 3 *Conus* species, 4 O-superfamily conotoxins formed monophyletic clades with several known pharmacological conotoxins, but the poor Posterior probabilities (0.30~0.69) at the nodes do not support related bioactivity prediction. The detailed functions of these high-abundance conotoxins in predation or defense deserve further investigation (such as by patch-clamp detection). Construction of electrophysiological platforms for the analysis of multiple neural ion channels and receptors is underway in our laboratory, which in turn proposes a potential hope for the development of novel conotoxin-based marine drugs for the treatment of neuroreceptor associated human diseases.

This study also identified many complete and partial sequences of 11 enzymes, which are potentially involved in the post-translational modification of conotoxins, as well as numerous functional proteins that may be related to the conotoxin biosynthesis processes including translation, protein folding, translocation, delivery and degradation. Gene Ontology (GO) functional classification (Supplementary Table S5) showed that conotoxin synthesis may be closely related to binding, catalytic activity, metabolic process and cellular process. The abundance of functional proteins underscores the fact that the venom duct is a metabolically active organ. Although there have been many reports on various novel conotoxins, our present work improves the understanding of conotoxin biosynthesis processes in vivo, which may provide insights into the fundamental mechanisms underlying the generation of complexly modified peptides in general.

## 4. Materials and Methods

### 4.1. Sample Collection, RNA Extraction and Sequencing

*C. caracteristicus, C. quercinus,* and *C. generalis* were collected in the offshore areas of Sanya City, Hainan Province, China. Specimen identification (using *COI* gene sequences [39]) was performed after they were collected and dissected on ice. Three intact venom ducts were separated and the total RNAs were extracted using TRIzol^®^ LS Reagent (Invitrogen, Life Technologies, Carlsbad, CA, USA) following the manufacturer’s instructions. Total RNAs were further treated with oligo-(dT)- attached magnetic beads (Invitrogen, Life Technologies, Carlsbad, CA, USA) to extract the mRNAs. Three non-normalized Illumina cDNA libraries were constructed separately and sequenced on the Illumina HiSeq2000 platform (Illumina, San Diego, CA, USA) by BGI-Tech (BGI, Shenzhen, China).

### 4.2. Sequence Analysis and Assembling

Raw sequencing reads from the 3 sets of transcriptome sequencing were cleaned up using SOAPnuke software (BGI, Shenzhen, Guangdong, China) [68] to ensure high quality for downstream analyses. Adapters and reads with over 10% of non-sequenced (N) bases or more than 50% of low-quality bases (base quality ≤ 10) were removed. Then the filtered reads were assembled into unigenes with SOAPdenovo-Trans v1.02 (BGI, Shenzhen, Guangdong, China) for *de novo* transcriptome assembling [69]. The FPKM value, a general parameter for quantification of gene transcription, was calculated for comparison [70].

### 4.3. Prediction and Identification of Conotoxins

All previously known conotoxins in the ConoServer database [19] were downloaded to construct a local reference dataset for conotoxin prediction from our 3 transcriptome datasets using the traditional homology search method. Subsequently, unigenes from each transcriptome were run against the local conotoxin dataset using Genewise v2.4.1 [71] and Agustus v2.7 [72] with an E-value of 1.0 × 10^−5^. Those unigenes with the best hits were translated into peptide sequences. A conotoxin generally consists of a highly conserved N-terminal signal peptide region, a less conserved intervening pro-peptide region, and a hypervariable C-terminal mature peptide region with conserved cysteine patterns [73,74]. A few conotoxins also have a post-peptide region at the C-terminal after the mature peptide region [75]. The predicted conotoxin transcripts were manually inspected using the ConoServer’s web-based ConoPrec and NCBI’s blastp. Those transcripts with duplication or truncated mature region sequences were removed.

### 4.4. Classification of Conotoxin Superfamilies

The distinct regions and cysteine frameworks of these predicted conotoxins were analyzed using the ConoServer’s web-based ConoPrec. Based on 75% identity in the conserved signal peptide sequences [57], these identified conotoxins could be assigned to most of the 27 known superfamilies in the ConoServer. The particular threshold values for I1, I2, L, M, P, S, con-ikot-ikot and divergent superfamilies were 71.85%, 57.6%, 67.5%, 69.3%, 69.1%, 72.9%, 64.5  ± 20.2%, and 64.22 ± 20.53%, respectively [33]. Those conotoxins without signal regions but still showing high similarity either in the proregion or mature region were considered as the “Unknown” group.

### 4.5. Annotation of Predicted Functional Proteins

Unigenes were firstly translated into amino acids in six frames and aligned with BLASTX to public protein databases (E-value ≤ 1.0 × 10^−5^) including NCBI non-redundant (Nr), Swiss-Prot [76], and Clusters of Orthologous Groups (COG) [77]. The protein with the highest sequence similarity was retrieved and annotated to each unigene. For the Nr annotation, Blast2GO v4.1 (Instituto Valenciano de Investigaciones Agrarias, Moncada, Valencia, Spain) [78] was used to determine GO annotation, which was defined by molecular function, cellular component, and biological process ontologies.

### 4.6. Phylogenetic Inference of Abundant Conotoxins

Bayesian analyses of the combined data were performed with MrBayes v.2.01 (University of Rochester, Rochester, NY, USA) [79] using the best-fit model indicated by Modeltest 3.06 (Brigham Young University, Provo, UT, USA) [80]. A Metropolis-coupled Markov Chain Monte Carlo algorithm running four Markov chains simultaneously was employed to estimate the posterior probability of phylogenetic trees. Each Markov chain was initiated with a random tree and run for 1,000,000 generations, sampling every 100 generations for a total of 10,000 samples per run. The first 2,500 samples of each run were discarded as burn-in, and the remaining samples were applied to construct a consensus tree using PAUP*4.0 (Florida State University, Tallahassee, FL, USA) [81].

### 4.7. Availability of Supporting Data

The datasets supporting the results of this article are included within the article and its supplementary files. The transcriptome reads generated in this study have been deposited in China National GeneBank Nucleotide Sequence Archive with accession numbers of CNS0048931 for *C. caracteristicus*, CNS0048933 for *C. generalis*, and CNS0048932 for *C. quercinus* under the project CNP0000360.

## 5. Conclusions

In this report, we have examined the diverse transcription repertoire in the venom ducts of 3 vermivorous cone snails, which are resident in the offshore waters of the South China Sea. We not only succeeded in characterizing the abundant conotoxin-encoding transcripts across 22 known superfamilies and 1 new superfamily, but also identified a variety of functional proteins that may be responsible for biosynthesis and delivery of these conotoxins. As expected, the majority of the identified conotoxins were novel, based on their transcript sequences, and some possessed new cysteine frameworks and divergent signal regions. Comparison analysis indicated surprising interspecific and intraspecific divergences in the conotoxin numbers and transcription levels, thus we made a primary conclusion that the abundant O-superfamily conotoxins in all 3 venom duct transcriptomes probably play major roles in the prey capture strategy of these vermivorous species. Our study with various cone snail species provides new insights into the complex biosynthesis mechanisms that lead to the remarkable variability of the venom composition. Our present work adds more conotoxins, which will definitely improve our genetic resource to develop new drugs.

## Figures and Tables

**Table 1:** Statistics of venom duct transcriptome sequencing data for the 3 *Conus* species.

Species	Raw Data (Gb)	Clean Data (Gb)	Q20* (%)	Nonsequenced (%)	GC Content (%)
*C. caracteristicus*	5.51	4.57	95.99	0	47.84
*C. quercinus*	3.47	3.21	98.31	0.01	47.3
*C. generalis*	5.32	4.37	96.00	0	47.09

* A quality score for the percentage of incorrect bases at less than 1%.

**Table 2:** Summary of sequences produced by the assembling for the 3 *Conus* species.

Species	*C. caracteristicus*	*C. generalis*	*C. quercinus*
**Clean reads**			
Total reads (n)	50,788,576	48,557,734	35,694,024
Base pairs (Mb)	4,570.97	4,370.2	3,212.46
Mean length (bp)	90	90	90
**Contigs (≥100 bp)**			
Total number	213,155	219,692	153,249
Base pairs (Mb)	47.84	60.75	40.22
Mean length (bp)	224	276	262
N50 (bp)	236	307	313
**Scaffolds (≥200bp)**			
Total number	79,324	103,682	61,926
Base pairs (Mb)	47.57	65.38	34.96
Mean length (bp)	599	630	564
N50 (bp)	794	891	717
**Unigenes (≥200 bp)**			
Total number	72,462	95,438	61,002
Base pairs (Mb)	39.61	54.87	33.67
Mean length (bp)	546	574	552
N50 (bp)	670	749	688

**Table 3:** Classification and cysteine patterns of the conotoxins identified from *C. caracteristicus*.

Superfamily		Number	Cysteine Pattern (Number of Conotoxins)
**A**		11	CC-C-C (8), CC-C (3)
**B1 (Conantokin)**		2	Cysteine free
**C (Contulakin)**		1	Cysteine free
**D**		2	C-C-CC-C-C-C-C (1), C-CC-C-CC-C-C-C-C (1)
**I**	**I1**	1	C-C-CC-CC-C-C
	**I2**	6	C-C-CC-CC-C-C (1), C-C-C-C-CC-C-C (4), C-C-CC-C-C (1)
	**I3**	4	C-C-CC-CC-C-C (3), C-C-CC-C-C (1)
**J**		7	C-C-C-C
**L**		4	C-C-C-C
**M**		6	CC-C-C-CC (5), CC-C-C-C-C (1)
**O**	**O1**	22	C-C-CC-C-C
	**O2**	11	C-C-CC-C-C (3), C-C-CC-C-C-C-C (3), C-C (5)
	**O3**	6	C-C-CC-C-C
**S**		3	C-C-C-C-C-C-C-C-C-C
**T**		9	CC-CC (8), C-C-CC (1)
**Y**		1	C-C-CC-C-CC-C
**Divergent M—L-LTVA**		1	C-C-C-C-C-C
**Unknown**		21	C-C-C-C-C-C (19), C-C-C-C (1), CC-C-C-C-C (1)
**Total**		**118**	

**Table 4:** Classification and cysteine patterns of the conotoxins identified from *C. generalis*.

Superfamily		Number	Cysteine Pattern (Number of Conotoxins)
**A**		2	CC-C-C
**B1 (Conantokin)**		1	Cysteine free
**C (Conotulakin)**		1	Cysteine free
**D**		1	C-CC-C-CC-C-C-C-C
**I**	**I1**	1	C-C-CC-CC-C-C
	**I2**	4	C-C-CC-CC-C-C (2), C-C-C-C-CC-C-C (2)
	**I3**	1	C-C-CC-CC-C-C
**L**		3	C-C-C-C
**M**		4	CC-C-C-CC (3), C-C-CC (1)
**O**	**O1**	12	C-C-CC-C-C
	**O2**	4	C-C-CC-C-C (3), C-C-CC-C-C-C-C (1)
	**O3**	3	C-C-CC-C-C
**P**		2	C-C-C-C-C-C
**S**		1	C-C-C-C-C-C-C-C-C-C
**T**		5	CC-CC
**Con-ikot-ikot**		1	CC-C-C-C-CC-C-C-C
**Conotoxin-like**		1	CC-C-C
**Divergent MSTLGMTLL-**		1	C-C-C-CCC-C-C-C-C
**Unknown**		13	C-C-C-C-C-C
**Total**		**61**	

**Table 5:** Classification and cysteine patterns of the conotoxins identified from *C. quercinus*.

Superfamily		Number	Cysteine Pattern (Number of Conotoxins)
**A**		5	CC-C-C
**B1 (Conantokin)**		3	Cysteine free
**I2**		3	C-C-CC-CC-C-C (2), C-C-C-C-CC-C-C (1)
**M**		10	CC-C-C-CC (9), C-CC-C-C-C (1)
**O**	**O1**	8	C-C-CC-C-C
	**O2**	3	C-C-CC-C-C
	**O3**	1	C-C-CC-C-C
**T**		1	CC-CC
**V**		3	C-C-CC-C-C-C-C
**Y**		1	C-C-CC-C-CC-C
**Con-ikot-ikot**		1	CC-C-C-C-C-CC-C-C-C-C
**Divergent M—L-LTVA**		2	C-C-C-C-C-C
**Unknown**		7	C-C-C-C-C-C
**Total**		**48**	

## Supplementary Materials

The following materials are available online at https://www.mdpi.com/1660-3397/17/3/193/s1. Table S1: Protein sequences of the putative conotoxin transcripts identified from *C. caracteristicus*. Table S2: Protein sequences of the putative conotoxin transcripts identified from *C. generalis*. Table S3: Protein sequences of the putative conotoxin transcripts identified from *C. quercinus*. Table S4: The top 10 conotoxins with the highest FPKM values in the 3 transcriptome datasets. Table S5: Conotoxin biosynthesis-related proteins identified from the 3 venom duct transcriptomes.
