# Supplementary material for: High-Throughput Identification and Analysis of Novel Conotoxins from Three Vermivorous Cone Snails by Transcriptome Sequencing
Source: Mar Drugs. 2019 Mar 26;17(3):193. doi: 10.3390/md17030193 (PMC6471451; doi:10.3390/md17030193)
Supplement: Supplementary file 1 [file marinedrugs-17-00193-s001.zip › Supplementary Table 1-459494.docx]

**Supplementary Table S1.** Protein sequences of the putative conotoxin transcripts identified from *C. caracteristicus*. ^1^

| A superfamily: CC-C-C; CC-C  1 MGMRMMFTVFLLVVLATTVVSSTSDHDA--ASNHENRRASNRINQRTWEECCKRPPCRVQHMGHC  2 MGMRMMFIVFLLVVLATTVVSNRAS-------NRENRRASNWN--TRMIDCCDIPRCYNERPEECREESSG |
| --- |
| 3 -----------------TVVPFTSDRASASRNAATDNKASELKALNARIPCCSYPACAQSNIDLCGGRR ^2, 3^  4 -----MFTVFLLVVLATTVVSFTSDRASEGRNAAAKDKASDLVALRVRG-CCAIRECRLQNAAYCGGI ^2, 3^  5 -----------------------SDRASASRNAATDDKASELKALNIRTVCCYYPTCAESNPYLCYGGR  6 -------------VLATTVVSFTSDRASDGRNAAAN--AFDLIALIARQNCCSIPSCWEKYKCS ^2^  7 --------------LATTVVSFTSDRE----SNHENRRASNRIPRAMWQECCADPPCRQNHMEHCPAN  8 -----MFTVFLLVVLATTVVSFPSDRGP--ASNHENSKGSNRNVWFTPDECCRDPPCREKILEYCLAGEAVAAAFGFRQLPYRLSSE  9 -------TVFLLVVLATTVVSFP---SE--ASNHENSKGSTRKGWYTINECCMDLVCREIIEA---MKKEFAAALDFRRLPYRLSSE |
| 10------------------------------ASNHENSKGSTRNGRHTIQECCMDPVCRLIIEK---FGKDFAAALDFRRLPYR |
| 11-----------------------------PASNHENSKGSTRNPWYTPEECCMDLVCRKIVR----FGKDFAAALDFRRLSYRQSSE |
| B1 superfamily(conontokin)：Cysteine free  12 --------------------------LLQKSAARSTDDNGKDRLTQRKRTLKKRGNMARGYEEDREIAETVRELEEAGK  13 -----------------------EPVLLQKSAARSTDDNGKDRLTQRKRFFKMRGNTARGLEEE--LEEYVREI  C superfamily(Contulakin): cysteine free  14 --------------------------------------------------MLTKFETKSARVKGLSFHPKRPWVL |
| D superfamily: C-CC-C-CC-C-C-C-C; C-C-CC-C-C-C-C |
| 15 MPRLEVMLLVLLIL------AGGQAVQGDGQGDGMDRYLQRGDRADYAMCFRHRPGSLWGRCCLTKMCGSACCNHSFCRCTHDKDMGHGCAC |
| 16 MPRLEVMLLVLLLLPLPHFYAGGHAMQGNGRGHGMDQSFLRSVRMS----QQRRS---WGPACQTRMCGGICCGSDSCGCK-----GNECDCP |
| I1 superfamily: C-C-CC-CC-C-C  17 MKLCVTFLLVLVILPSVTGEKSSERTRIGAVLKGHWCGYPGERGCRYHSQCCGDMCCYDRKCVVTAMPC |
| I2 superfamily: C-C-CC-CC-C-C; C-C-C-C-CC-C-C; C-C-CC-C-C |
| 18 –MFGHTSVRFLLLSIMLLGMAEMVLCTCDSEFG-ETCEQ--EKVCSCSNHGCCEAASSAKTDQCMTQGACVAWLSDNVKRRVIQKQKRFLGMVRGLA |
| 19 –MFGHTSVRFLLLSIMLLGMAEMVLCTCDSEFG-ETCEQ--EKVCSCSNHGCCEAASSAKTDQCMTQGACMAWLSDNVKRRAIQKQKRFLDMVRGLA  20 –MFGNTSVSFLLLSITVLGMVATVICSCKYGEWTEKCGQPGE-TCECPNHVCCDLK----RDVCMPSKSCKALIHKIVFRRSTRV  21 ---------FLLLSITVLGMVATVICSCNGRISSENCEQPGEQICSCPNHECCHFHP-PKRDQCMTRGMCYMALAGMPGRRSTQMQERFLRIPRGLAE  22 MMCRLTSLCFLLV-I-VLLNSAVDGIPCHGPGGWCSTHMWCCDPHNVCCDFPGAARCTRKRECSWPTIPQGRLAQLSHFFRR |
| 23 ----------------VLLNSAVDGIPCGGTGGFCYWLMPCCDSRDFCDRSVCTRESDLVVHITRGRRALHTRFFRR |
| I3 superfamily: C-C-CC-CC-C-C; C-C-CC-C-C |
| 24 MKLVLAIVVILMLLSLSTGAEMSDNHASMSANALRDRLLGPKALLCGGTHARCNRDNDCCGSLCCFGT---CISAFVPC ^2, 3^  25 ------IVVILMLLSLSTGAEMSDNHASRSATALIDRLLHQRASICYGTGGRCTKDKHCCGWLCCGGPSVGCVVSVAPCK ^2, 3^  26 --------VILMLLSLSTGAEKSGLEISMVGPPLYFWSPSP---ACKLSGEDCGYGYSCCEDLSCRPLIEPDTLKITALVCQIESA  27 -------------------------HASRSATAWRDRHLSPKALLCGGVRASCSRHDDCCGSLCCFGTSTGCRVAVRPCW ^2, 3^  J superfamily：C-C-C-C  28 MASVQSVACCCLLWLMLSVQLVTPGSPGTAQLSGHRTARVPAPHIMVPIYLCPILCKVGGVNTPFCNCTKKREMVSS  29 MASVQSVACCCLLWLMLSVQLVTPGSPGTAQLSGHRTARIPI--------VCSLLCKAGISIPIFCNCTKKRDVVS  30 MASVQSVACCCLLWLMLSVQLVTPGSPGTAQLSGHRTARIP---------VCSVLCNAGVDVP-FCDCTKKREMVSS  31 MASVQSLACCCLLWLMLSVQLVTPGSPGTAQLSGHRTARVPAPHIMVPIYLCPILCKVGGVNTPFCNCTKKREMVSS  32 MMSVQSVTCCCLLWLMLSVQLVTPGSPGTAQLPRDRTARVSAES--VLEIICPYLCPLGIGALSFCNCPNKRDVVSSRITSRKRSMAV  33 -----------------------------AQLPRDRTARVPAEP--ILELICPHMCRLGIGEPPFCKCRNKRDVVSSRITSRKRSMAM  34 -----------------------------AQLPRDRTARVPAQP--VLDIICPGMCLLGIGEP-FCNCSNKRDVVSSQITRRKRSMAV  L superfamily：C-C-C-C  35 MKVSVTFIVVLMLTTSLTCGLSNGETHDPDAADQLVREERASRACNPPCTGNAMCQNGHCGYIRF  36 MKLSVMFIVFLMLTMPMTGDGNNRRAANGGEVGMPAYERAAKLLALLRERQCPAPCYPNCEEC  37 MKVSVTFIVLLTLTATLSHGFILRAIDGRENLGGWGNSQVENRQDYQIQTRGCPEDYPCKAGYVCDKGICRPLVIM  38 --------VFLMLTMPMTSDANNRHAANGGEAGELAGDRAAKLMALLQERQCGKHCPTCRHC  M superfamily：CC-C-C-CC; CC-C-C-C-C  39 MMSKLGVLLTICLLLFPLTAVPLDGDQPADRPAERK-QDVSSEQ--HPFFDPVKR--CCRR--CYMGCIPCCF ^3^  40 –MLKMEVVLFTFLVLFPLSALQLETDQPVERYAENK-QDLNPDESRNFMLPIVKK--CCTA--CRMPPCTCCA ^3^  41 ---------------------------PVERYAENK-QDLNPDERKDFILPIVR--GCCTA--CHIPPCTCC  42 ----MGVVLFTFLVLFPLATLQLDADQPVERYVENK-QDLNPDDRMGFILPALRRYECCVWPHCDGGCSSCVRSCE ^3^  43 -----GVVLFTILVLLPLATLLLEADQPVERQQDLNPQRGTRGIMKHVMSKGMSRRGCCTGQGCWNVPICECC ^3^  44 –VLKMGVVLFTFLVLFPLATLQLDADQPVERYAENK-QDLSPDKRVEFILHALGQRFCCVFPWCTGCYCC ^2, 3^  O1 superfamily: C-C-CC-C-C  45 MKLTCVSIVAVLFLAACQLITADDSREKQGYSAVRSSDKIQD--SDDLELTKGCMEDGDVCELGNH---NCCSGSCLGFEDEGICAVGVEYY  46 MKLTCALIVAMLLLTACQLTTADASRGRQEYPTKRLRAKMLN--SKFIKLIKRCAAPGASCSKYDN---ECCDACLLQYPNPPVC  47 MKLTCVVIVAVLFLTACQLNDADDSRYKHETRLVTLFRRRRE--SDSGLAPGTCVLFGSMCKAKTAS--ICCYKCDLEEGIC  48 MKLTCVIIVVALFLTACHAKG------KQEYPAVRGSDEMQD--SEDLKLAKKCTVDSDFCDPGNH---NCCSGKCIDEGGSGVCAIIP  49 MKLTCVLIVAVLFLTACQLIAADDFRDLQKFPRRKMSDRMPN--TKGV--ERDCLPPLTWCSMTDD---ECCNDCVLFLC  50 MKLTCVLIVAMLFLTACQLIAADDYRDLQEFPRRKMSDVILN--TKDT--EKRCLPGTATCNLYNN---LCCNYCLIFWC  51 MKLTCLLIVAMLFLTACQLATADDSRDKQEDHLLRSHREKQK--SEDPKMAVRCSYFGADCLPDSH---DCCSGECFGFPDMGLCT  52 MTMTCVLIIAVLFLTACQLITADYSRDKQENPVERSRIKMIK--SWRPKLNKRCTNPGGYCVVPHHN--ECCSGQCDPSSIIGQC  53 MRVTCVLIIAVLFLTACQLITADYSRDKQENPVERSRIKMIK--SWRPKLNKRCTNPGGYCVVPHHN--ECCSGQCDPSSIIGQC  54 –RVTCVLIIAVLFLTACQLITADYSRDKQENPVERSRIKMIK--SWRPKLNKRCTNPGGYCVVPHHN--ECCSGQCDPSSIIGQC  55 --LTCMMIVAVLFLTAWTFVTADDTRDGLKNLFPKARLEMKN--SEASRSRGRCRPPGMVCGFPKPGP-YCCSGWCFAVCLPV ^2, 3^  56 ----CLLIVAMLFLTACQLATADDSRDKQEDHLLRSHREKQK--SEDPKMAVRCSYFGADCLPDSH---DCCSGECFGFPDMGLCT  57 -----VVIVALLFLTACQLITADNSKGTQKHRALRS--T-----TKLSMLTRGCTPPGGACGYHGH----CCDFCDTFGNLC  58 ---------AVLFLTAWTFVTADS-IRALEDLFAKARDKMEN-SGASPLNQRDCKALGEFCGIPYVHNSQCCSQLCGFICV  59 ------------FLTAWTFVTADDSINALEDLFSKARDEMEN-GEASTLNERDCKADGAFCGIPFVKNWMCCSNLCIFACVPE  60 ----------------CQLNTADDSRDEREYRAVRLRDAMRN--FKGSK--RDCGAQGEGCYTRP-----CCAGLSCVGGHSGGLCQY  61 ----------------CQLNTADDSRDKQEYRAVRLRDGMRN--FKGSK--RNCGEQGEGCATRP-----CCSGLSCVGSRPGGLCQY  62 ----------------CQLITADDSREKQGYSAVRSSDKIQD--SDDLKLTKRCTENGDVCDPENH---NCCSGSCLDDEDPPVCGF  63 ------------------------------KLFSKARDEMKNPEDSKLDKKKVCLESGAVCVIPIIASFACCSAFCYGVCL  64 -----------------------------------------T--DKNSKLTRQCSPNGGYCTLHIH----CCSNHCIKPIGRCVA  65 ----------------------------------------ST--DKNSKLTRQCSANGGSCTRHYH----CCSLYCNKDSSVCVATSYP  66 --------------------------------------------DKNSKLTRQCSPVGGSCSHHYN----CCSLYCNKNIGQCLATSYP  O2 superfamily：C-C-CC-C-C; C-C-CC-C-C-C-C; C-C  67 MKKLTILLLIAAVLMLTQALIQ---EKRPEDEIKFLSKRKSGAQRWWDGECRLWSNGCRKHKECCSNHCKGIYCDIW ^2, 3^  68 MEKLTILLLVAALLMSTQALIQGGGEKRQKAKINFLSKRKSTAESWWEGECSGWSVYCEYDSECCSGECGGYYCELW  69 MKKLTILLLVAALLMSAQALIQGGVEKRQKAKMDF-SKRKTTAEKWWDGDCMTWYAPCRLDSQCCSGNCGKHRCLAW  70 MEKLTILILVATVLLAIQVLVQSDREKPLKGRVKQYAAKRLSALLRGPRECTPMHRACEEDEECCPNLECKCSNNPDCQSGYKCRP ^2, 3^  71 MKRLTILILVATALLSTQVMVGGDGE---KPLMRKNAANRLLAPMRGKR-CKTEDYLCQKDEDCCSGFDCRCTVNANCSPPHIRCKP  72 --------------------------------------------------CRVENLCPHTVCCDRSRCSCKLIRTRPLMYHVCVC  73 ----------------TQVMVQGDADQPADRDAVPRDDNPGGTSGKSMNALRQPGCRWWSRWC  74 ----------------TQVMVQGDGDQPARRDAVPRDDNPGGTSGKFMNVLRQYGCPPGLWCG ^2^  75 -----------------QVMVQGDGDQPAARNAVPKDDNPGGEAGKFMNVLRRSGCPWEPWCG ^2^  76 -------------------------DQPAVRDAVPRDENPGGTSRKFMNALRRNGCPPGLWCG ^2^  77 MEKLAVLVLVAAVLLSTQVMFR---DQPADRDAVPRDDNPGGMSGGFMNVRRRSGCPWHPWCG ^2^ |
| O3 superfamily: C-C-CC-C-C  78 MSGLGMMVLTLLLLVFMETSHQDAGEKKATQRDAINVRRRRSLAQRTVTEECEESCEDEEKHCCNTNNG-PSCARICFG  79 ---------------FMETSHQDAGEKQSTQRDAINVRQGRSLTRRKVPEACEESCEEEEKHCCNTSNG-LSCVRHCLG  80 -------------------------EKQAMERDAINIRWRRSLTRRIVTEECEEYCEDEEKDCCGLQNGQPFCAPFCLG  81 ---------------------------------AINVRQRRSVTRRVITEVCEGYCEVLRKHCCGTRDAIPVCNSACRG |
| 82 ----------------------------------INVRRRRSLTRRVVTEACEETCEDEEKTCCGLENGEPVCARFCLG |
| 83 ---------------------------------------RRSLAQRAVTEECKEDCNDENKKCCGEENGEPVCATACLG |
| S superfamily： C-C-C-C-C-C-C-C-C-C  84 MMLKMGAMFVLLLLFILPSSQQEGDVQARKTHLKRGFYGTLAMSTRGCSGTCHRREDGKCRGTCDCSGYSYCRCGDAHHFYRGCTCSCQG ^2, 3^  85 MWKMGVMLCVVFLLSPLASLRQEGEVQARKIDLKSNLYGAWKRPARDCRGTCG--STDRCTGTCECEGYESCSCSNSGQ-HSGCTCSC  86 ------------------------------------------MSTRGCSGTCHRRQNGECQGTCDCDGHDHCDCGDTLGTYSGCVCIC |
| T superfamily: CC-CC；C-C-CC  87 MRCLPVFIILLVLIASAPSVDARPQTK-DDALASFHDSAKRHLQRLVNARKCCPESPPCCHYYYYFGRRK  88 MRCLPVFIILLLLITSAPSVNAKLKTK-DVRLLPFHDNAMRTLQRLWKKCLGCGEDPRCC  89 MRCLPVFIILLLLIPSASSVDAQPKPKNDLLLAPFHDNAKRTLQLLRNKRICCPWFALCCYY |
| 90 MLCLPVFIILLLLASPAAPNPFETRLLSDLTRADADMETEKYLGEVR-KAVCCQVFPGSDCCPG ^2, 3^  91 --------------SPAASDPLEKRIQNDLIRAALEDADVENDPRILTPIINAAKLGCCKLFDVGFCCGKK  92 --------------------------------AALEDADTKNDPRILEDIVSTALATCCKFQFLNFCCNEK ^3^ |
| 93 ---------------------------------ALEDADTKNDPRLIGGLISTALAACCKVKFLNFCCNEK |
| 94 ---------------------------------ALEDADTKNDPRLVGTLVSTALTVCCQFEFLNFCC |
| 95 -----------------------------------------------GILQEHWNKRCCPRRLACCIIGRK ^2^ |
| Y superfamily：C-C-CC-C-CC-C  96 MQKATVLLLAILLLLPLSTAQDAEGSQEDAAQREVDIATRCGGTGHSCNEPAGELCCRRLKCVNSRCCPTTDGC  geneSuperfamily=Divergent M---L-LTVA: C-C-C-C-C-C  97 -----LVTVALLLTFVMSIDSAPADQTETGRVSLREDQRFPCSSGRCACLPKDGSSTSFQCQSTSASTENCFDNHCITEDEW  Unknown superfamily: C-C-C-C-C-C；CC-C-C-C-C；C-C-C-C  98 -----VLILAICMLALGSGATRRRAVRSVCTLQKDTGPCKMAIPRYYFNMDISDCDTFIYGGCFGNANNFETYEECDDTC  99 -----VLILTICMLAVAAGASRR---WGLCSLPAEAGPCYASITRYYYDRKTQECTQFYYGGCGGNSNNFDTAEECDDVC  100 ----VLLLVTLCVQ-AEDK---------CQQPKAPGRCMAYMERYFFNSEKGACEQFIYGGCEGNENNFETLEACQTAC  101 -----LLLTATVIVVATGNR--------CRLPSDTGPCRAAIRQFYYNWTERQCQDFIYGGCGGNDNRFETREECERAC  102 ---------------KIDN---------CTLPAERGPCMANLTMYFYNWTSEQCEEFNYGGCGGNPNNFHNMTECEATCSR  103 ---------------------------VCKQAPSPGRCNAVFRRWYFNVHVAACSWFTYSGCGGNDNNFRSREECERMC  104 ---------------------------ICQLEADVGPCSGTFPRWFYNSDMRKCQLFDYGGCRGNDNRFDTEEECMELC  105 -----------------------DFVSICDMPEDPGPCRGRLPRWFYDPLDRQCRAFYWSGCQGNENNFLTVQECQQTCM  106 --------------------------DICRMPKVVGPCMAGITRYYYDTASAACRQFIYGGCQGNLNNFGSLEACQGKCARH  107 ----------------------------CQLPKDPGPCTSPIHRFFFNSETGACEVFIWGGCYGNANKFKTLEECQETC  108 --------------------------DRCHLPPETGMCRAYMPMYFYNATLGRCQGFIYGGCNGNDNKFNTEEDCMKAC  109 ---------------------------VCSLPRERGPCSNYEIVWYYDTEEERCKRFYYGGCQGNGNRFANREECEGRCVR  110 ----------------------------CNLPQIVGPCKAYMPSFFYNTGTGQCERFVYGGCGGNANRFETKQECQGQCQR  111 --------------------------DPCQLPKDPGPCPNQHHNFFSDSEMGACKMFIYGGCYGNANNFRTLEECQATC  112 --------------------------EICQQPRQVGPCRAAFRRWFYNKFTRTCEQFIYGGCKGNGNNFQSLPECQDRC  113 -----------------------DFVSICDMPEDPGPCRGRLPRWFYDPLDRQCRAFYWSGCQGNENNFLTVQECQQTCM  114 --------------------------DICRMPKVVGPCMAGITRYYYDTASAACRQFIYGGCQGNLNNFGSLEACQGKCARH  115 --------------------------DLCFQPMVVGLCKASFPNYYYNPALGTCQLFYYGGCGGNKNRFGTKDACLKTC  116 ---------------------------ICQLEADVGPCSGTFPRWFYNSDMRKCQLFDYGGCRGNDNRFDTEEECMELC  117 -----------------------RYVENKQDLSPDERMGIRSTVTSPEGQKCCLSGFCDGACRQCKDTCE  118 --VFNKAKDKFNELTSGLGVHFDRIVDLLIDQIDSGMTEAACIKVCESSASKLLGDASPMAGTVCAPVCTAALAKLEEVA |

^1^Signal regions are shown in red, mature regions are shown in green, and cysteine residues are marked in bold black. The sequences isolated and identified previously are highlighted in grey shading. ^2^Mature peptides in the sequences were aligned with previously recorded conotoxins in ConoServer. ^3^Mature peptides in the sequences were aligned with previously recorded conotoxins in GenBank.
