# Supplementary material for: High-Throughput Identification and Analysis of Novel Conotoxins from Three Vermivorous Cone Snails by Transcriptome Sequencing
Source: Mar Drugs. 2019 Mar 26;17(3):193. doi: 10.3390/md17030193 (PMC6471451; doi:10.3390/md17030193)
Supplement: Supplementary file 1 [file marinedrugs-17-00193-s001.zip › Supplementary Table 2-459494.docx]

**Supplementary Table S2.** Protein sequences of the putative conotoxin transcripts identified from *C. generalis*. ^1^

| A superfamily: CC-C-C |
| --- |
| 1 MGMRMMFTVFLLVVLAINVVSVTSYRASHGR-----KDAADLSALNDNNN**CC**NHPA**C**AGKNSDL**C**G  2 --------VFLLVALATTVVPFTSDRASASRNAATDNKASELKALNARIP**CC**SYPA**C**AQSNIDL**C**GGRR ^2, 3^ |
| B1 superfamily(conontokin): cysteine free |
| 3 MQLYTHLYLLVPLVTFHLILGTGTLAHGDALTERRSADATALKPEPVLLQKSAARSTDDNGKDRLTQMNRILKKRGDTARGLEEDLELRLLENSKKQENEK  C superfamily(Contulakin): cysteine free  4 ---------------------------------------------MLTKFETKSARVKGLSFHPKRPWVL |
| D superfamily: C-CC-C-CC-C-C-C-C  5 MPKLEMMLLVLLILPLPYCNAAGVTTVQWGGHGDGLDRYLQRGVRDVHRP**C**QSVRPGRVWGK**CC**LTRL**C**STM**CC**ARAD**C**T**C**VYHTWRGHG**C**S**C** ^2, 3^  I1 superfamily: C-C-CC-CC-C-C  6 MKPSMTLLLILMILPSMTGEKSRERRLSSAAVKGFLRPAR**C**TEFGKE**C**NLPPE**CC**GRM**CC**YNSL**C**MWSSKE**C**  I2 superfamily: C-C-CC-CC-C-C; C-C-C-C-CC-C-C  7 –MVGHTAV-RFLLLSILLLHMAAMVSCD**C**DYDYGKT**C**EGGEA**C**E**C**SNHI**CC**DVLSQAKKDQ**C**VRGHDV**C**YLLNQSSRRRRAIQMQKRSRGMLRGLA  8 ------------------LGMVATVICS**C**ESGVSGET**C**DLSVEKR**C**S**C**SRHI**CC**THHALEQHK**C**MTWAK**C**MSVSLGVNGRRSIQMQDRFLRMLRAFDD  9 -------VGCFLLVIVFLNLAGVQVVLGGA**C**RLEGMF**C**IHSPE**CC**LQD**CC**SGI**C**NSGA**C**GKRAQHQRLHLRRF  10 MMFRLTSVGCFLLVIVFLNLV---VLT-DA**C**TDEGEY**C**TDDLQ**CC**KLQ**CC**RAS**C**SDI**C**RFPGKRMHGHGLLRFFGQR  I3 superfamily: C-C-CC-CC-C-C  11 MKLFLVIVLNLMLLSLSTGAETSDNRASRSATALRDRLRRPKR**C**RNRFEE**C**WEDSE**CC**EEL**CC**DGPNY**C**KDSIGR**C**H  L superfamily: C-C-C-C  12 MKLSVMFIVFLMLTMPMTGDGNNRRAANGGEVGMPAYERAAKLLALLRE-RQ**C**PAP**C**YPN**C**EE**C**  13 MKLSVTFIVVLMLTTSLTCGFSLPSNKEERAFGPRDPDAADQLVREERASRA**C**NPP**C**TGLSM**C**QAGR**C**GYIRFR  14 MKLSVTFIVVLMLTTSLTCGFSPPSNNGERAFGSHDPDAADQLVREVRASSA**C**PEP**C**AKGST**C**IGKT**C** |
| M superfamily: CC-C-C-CC; C-C-CC  15 -MMKMGAMQFTFLVLFLLVALQVDADQPAERHAENKQDLNPVERKEIIVPALRQNTNSDEED**CC**IYPW**C**DGD**C**L**CC**  16 MMFKLGVLLTICLVLFPLTALPLDGEQPVDRHAEHMQDDNSAAQNPW-VIAIRQ--------**CC**TS--**C**NFG**C**QP**CC** |
| 17 -MSKLGVALLIFIVLFPLANLQLDGDQPADRHAEERRGLLHELYKLLRRSQTRVKEPEELRVE**C**PIYY**C**PDSV**CC**KK  18 –MSKLGVVLFTILVLLPLATLLLEADQPVER----QQDLNPQRGTRGIMKHVMSKGMSRRG-**CC**TGQG**C**WNVPI**C**E**CC**V ^3^ |
| O1 superfamily: C-C-CC-C-C |
| 19 MKLTCVLIIAVLFLTACQLITGE-----------QKDHALRSTDKNSKLTRQ**C**SPNGGY**C**TLHIH**CC**SNH**C**IKPIGR**C**VA |
| 20 MKLTCVMIVAVLFLTACQLTTAGDSRDKEG----YRAVRSIPSMQDGIDSRE**C**RRRGQG**C**TQSTP**CC**DGLR**C**DGQRQGGM**C**VDS ^2, 3^ |
| 21 MKLTCVLIVAVLFLTVCQLIPADYSRDKPGYPAWKLRTKMQN-SRRSKLARS**C**KERASS**C**ETPSE**CC**SGV**C**RTRIFYL**C** |
| 22 MKLTCALIVAMLLLTACQLITTDDFRGRQQYRTARARTKMQN-YKIFRLTKR**C**DAPNAP**C**EKFDND**CC**DA**C**MLREKQQPI**C**AV ^2, 3^  23 MKLTCVLIITVLFLTACQLTTAVTYSRGE-----HKHRALMSTGTNYRLPKT**C**RSSGRY**C**RSPYD**CC**RRY**C**RRITDA**C**V ^3^  24 MKLTCVLIVVVLFLTACQLIPADYSRDTPGYPAWKLKTKMQN-SRRWKLAKR**C**KGKGAG**C**DYSHE**CC**SRQ**C**TGRIFQT**C**N ^2, 3^  25 MKLTCMMIVAVLFLTAWTFVTADDTRYRLENPFLKARNELQKHEASQLNERG**C**LDPGYF**C**GTPFLGAY**CC**GGI**C**LIV**C**IET ^2, 3^  26 MKLTCVLIITVLFLTACQLTTAVTYSRGK-----QKHRALRSTDKTSWLTKP**C**SRLMEP**C**TQHPQ**CC**SNT**C**SKFTTK**C**IS  27 MKLTCMMIIAVLFLTVWTFVANDDPRNGRQNRFSKARHGMKNRGAHRLDQRV**C**IKDGEF**C**GTPVENDPL**CC**SLI**C**DLV**C**L  28 ------------------------SRDKEG----YRAVRSRLGVRHGMRFSE**C**STRG--**C**TFSSH**CC**EGLR**C**QGTPQGGV**C**V  29 ----------------------------------KLRTKMQN-SRRSKFART**C**RERGIG**C**DDPLE**CC**SHQ**C**TFTGFST**C**  30 ---------------------------------------MQN-SRRSKLARE**C**LPKGAG**C**DEPFE**CC**SHL**C**TGRLHTT**C** |
| O2 superfamily: C-C-CC-C-C; C-C-CC-C-C-C-C |
| 31 MEKLTALILVATVLLTTQVLVQSDGERPLKRRVKEYAAKRLSALMRGSRQ**C**LPQYHP**C**IIGNEE**CC**PHLE**C**K**C**LPKP |
| 32 MEKLTTLILVATVLTTIQVLVKSDRERPPKRTVEQYAAERLLALMRGSRQ**C**KPKGDI**C**EE-DEE**CC**PSLE**C**E**C**DSLPT**C**ITNKR**C** |
| 33 MEKLTILLLVAAVLLSTQVLVQCDGEKP-KKTKLRFLKARMSSREE---P**C**KERRQP**C**ER-NEE**CC**NDS-**C**NRFY**C**  34 –QKLTILLLVAAVLISTHILGQGDGEKR-MKTKMDFFKARKHWAYKQARD**C**KGWLEE**C**EEEN--**CC**DPYE**C**IGF**C** |
| O3 superfamily: C-C-CC-C-C  35 MSGLGIMVLTLLLLVSMATNHQDG-----------RVRRLMLRNRLRQMM**C**TTDED**C**PTGQE**CC**PDNINDPQGF**C**VDD**C**IV |
| 36 MSGLGIMVLTLLLLVFMETSHQDAGEKQATQRDAINVRRRRSLTRRVVTEA**C**EES**C**EDEEKT**CC**GLENGEPV**C**ARF**C**LG  37 ---------------------------------------LMLRNRLRQMM**C**SSNED**C**PAGQE**CC**PDNLGKPEGF**C**VDD**C**II  P superfamily: C-C-C-C-C-C  38 --------------LLFALGTFVGVQLEQITRDVDNGQPTDNRHNLQSVWKQMSLLRSVVKR**C**VGS**C**DLNNP**C**SSG**C**I**C**NGDK**C**  39 -------------------GNFVGVQPGQITRDMENGQLMDNRRDLRSPWKQMSLFRSFKLV**C**GKG**C**KSRPD**C**PTE**C**F**C**YMDM**C**  S superfamily: C-C-C-C-C-C-C-C-C-C  40 ----------KVGLPFVLLLLLTSTSPKQERDVQARKRSLNSDLYRSLARSTRG**C**GGT**C**YDSQH**C**DGT**C**Y**C**PAGD**C**Y**C**GTEGPHSG**C**T**C**I**C** |
| T superfamily: CC-CC |
| 41 MRCLPVFIILLLLVPSAVSVDVQPETKNFMTLVSRDFAKKSLKGLSNK-RD**CC**QRNFL**CC**  42 MRCLPVFIILLLLIASAPSVDAQPKTKYNAPLTSLHDNAKGILQEHWN-KR**CC**PRRLA**CC**IIGRK ^2^  43 -------IIVLLLVPSAHGIDAEAKTKSDESHASLHDKAKGILQRLSSSRG**CC**PNHSS**CC**  44 ----------LVLIASAPGVDARPQTK-DDALASFHDSAKRHLQRLVNARK**CC**PESPP**CC**HYFGRRK ^2, 3^  45 --------------------------------------RILEDIVSTALAT**CC**KFQFLNF**CC**NEK ^3^  Con-ikot-ikot: CC-C-C-C-CC-C-C-C  46 MATNMLMTLSVFVMVVMAATVVGSTPLPEPELSRSVRDSRT**CC**IDSTLQ**C**LRGYPGEEYTYATM**C**NLEASGP**C**GLSVYQG**CC**NGYMN**C**IR  INVGNLRLEGAHNA**C**KNRR**C**  conotoxin-like: CC-C-C  47 MRCLASLVVTLLLFTATATTGASNHVNAAASGKASDSISLAARDD**CC**PNPS**C**RQNHPER**C**  Divergent MSTLGMTLL-: C-C-C-CCC-C-C-C-C  48 MSTLGMLLLIALLLPLTNPADNGDGQAKPRSRNLRSLDFMRTHRRLDKRG**C**DPTDG**C**KKAL**C**NTDTGP**CCC**QHGHN**C**QTQPSGRRA**C**VRN**C**PH  N**C**P  Unknown superfamily: C-C-C-C-C-C  49 ----------------AVCVTLVGKKPTVDV**C**SLPADPGP**C**EALDRRFFFDKVDGT**C**KPFNYGG**C**QGNGNRFDSKSR**C**ERA**C**  50 -----------------------------DV**C**ALPKVTGP**C**FAAFPRFYFDQTAGR**C**KTFTYGG**C**HGNQNNFRSLRA**C**RNT**C**A  51 -----------------------------EV**C**SLPRERGP**C**SNYEIVWYYDTAEQR**C**TRFYYGG**C**QGNGNRFANREE**C**EER**C**VR  52 -----------------------------DL**C**YQPMKVGP**C**RSKVP**C**YYFDHEYGK**C**QLFYYGG**C**RGNDNRFETKDA**C**LHT**C**  53 -------------------------------**C**QLEPDTGL**C**RAAFRRFYYNWNEQQ**C**QAFIYGG**C**GGNENRFKSREE**C**EQA**C**  54 -------------------------------**C**NLPKETGP**C**RALDHSFFYDVNAGQ**C**KHFIYGG**C**GGNANRFKTMAE**C**KWS**C**A  55 ----------------------------DA**C**SLPLSTGK**C**EQQQTRWHYNYRSGS**C**EKFIYTG**C**LGNANNFPTADA**C**QAR**C**  56 -----------------------------I**C**QLEADVGP**C**SGTFPRWFYNSGMRK**C**QLFDYGG**C**RGNENRFDTEEE**C**MEL**C**  57 -------------------------DFVSI**C**EMPEDPGP**C**RGRLPRWFYDPLDRQ**C**RAFYWSG**C**QGNENNFLSVQE**C**QQT**C**M  58 -----------------------------V**C**SLAPETGN**C**RANIPRWYYDAQFGQ**C**RQFVYGG**C**RGNSNNFETEQD**C**LNY**C**RR  59 -------------------------DFVSI**C**EMPEDPGP**C**RGRLPRWFYDPLDRQ**C**RAFYWSG**C**QGNENNFLSVQE**C**QQT**C**M  60 -----------------------------L**C**RLPAVPGP**C**RSRQPRYFYNYKVGK**C**QRFNYGG**C**KGNTNRFLTLGE**C**QSR**C**  61 ----------------------------DI**C**RMPKVVGP**C**RAGITRYYYDSASAA**C**RQFIYGG**C**QGNLNNFGTLEA**C**QGK**C**ARH |

^1^Signal regions are shown in red, mature regions are shown in green, and cysteine residues are marked in bold black. The sequences isolated and identified previously are highlighted in grey shading. ^2^Mature peptides in the sequences were aligned with previously recorded conotoxins in ConoServer. ^3^Mature peptides in the sequences were aligned with previously recorded conotoxins in GenBank.
