# Supplementary material for: High-Throughput Identification and Analysis of Novel Conotoxins from Three Vermivorous Cone Snails by Transcriptome Sequencing
Source: Mar Drugs. 2019 Mar 26;17(3):193. doi: 10.3390/md17030193 (PMC6471451; doi:10.3390/md17030193)
Supplement: Supplementary file 1 [file marinedrugs-17-00193-s001.zip › Supplementary Table 3-459494.docx]

**Supplementary Table S3.** Protein sequences of the putative conotoxin transcripts identified from *C. quercinus*. ^1^

| A superfamily: CC-C-C; CC-C-C-CC |
| --- |
| 1 MGMRMIFTVFLLVALATTVTS---DRVSNGRKAAAKFKAPALMELSVRQGCCSDPACAVSNPDICSGGR |
| 2 MGMRVMFTLFLLAVLSTTVVSFTLDRASNGRDAAADSKAADQIAQTVRDECCSNPSCAQTHPEIC ^2, 3^ |
| 3 MGMRMIFTVFLLVALATTVASFTLDRASNGRNAAADDKPSDWIALAIKQ-CCANPPCKHVNCR ^2, 3^ |
| 4 ----------------------TSDHASDGRNTAANDKASKLMAL--RNECCDNPPCKSSNPDLCDWIS  5 ---------------------------SDGRNTAANDKASDLMAL--RDGCCSNPSCSVNNPDICGGGR ^3^  B1 superfamily(conontokin): Cyteine free  6 MQLYTHLYLLVPLVAFHLILGTGTLAHGDALIERRSADATALKPEPVLLQKSAARST---DDNGKDRLTQMKRILKKRGNTARGYEEDREVAETVRELEVAGKRKRLI  7 MQSFTCMCLLVPLLFFHLAQRSDTAGHGGAATDVRSADHTLKRFLHDFRRAPKRRSNGRYDRRQSTSVGLAKEILAERLHEGQEERAEDSREASLEKLREIGR  8 MQSFTCMCLLVPLLFFHLAQRSDTAGHGGAATDVRSADHTLKRFHHDFRRAPKRRSNGRYDRRQSISVGLATEILAEGYR-------EDSREAAVEKLQEIGR  I2 superfamily: C-C-CC-CC-C-C; C-C-C-C-CC-C-C  9 -MVGRTSVSFLLFSIMVLGMVATVICSCEERISSEKCEAPGERICSCSNHVCCQLSAAKKDQCMTPFMCRTASGGNRRRRSTQVQDRFLRMARGLAD  10 MMFRLASVSCFLLVIVFLDLVVLTNACYPEGDYCEYHYQCCKGSCCFAYCRDPCRRVGKKAQLQEFLRHR  11 -------------------LVVLTNACYPEGEYCEYNYQCCESSCCVNYCRYPCRKRAQLQELLR |
| M superfamily: CC-C-C-CC; C-CC-C-C-C  12 -MLKMGVVLFTFLVLFPLATLQLDADQPAARYAENKQDLNPNERKKTMLSALRQRACCDPHWCDAGCYDGCC |
| 13 -MLKMGVVLFIFLVLFPLATPQLDADQPLARYAENKQDLNPNERMKIMLSALRQRACCEPLWCDAGCYDGCC  14 –MLKMGVVLFTFLVLFPLATLQLDADQPVARYAENKQDLNPNERMKIMLSALRQRECCEPSWCDAGCTDGCC ^2^  15 -------------------------------------------RMKMMLSALRQRGCCEPFWCDSGCTEGCC |
| 16 MMYKLGVLLTTCLLLFPLTAVQLDGDQPVDLPALRTQDFAP-EH--SPWFDPVKR-CCSQDCLVCIPCCPNGS |
| 17 MMSKLGVLLTICLVLFPLTALQLDGDQPADRPAERTQDISS-EQ--YRKFDQRQR-CCRWPCPGSCRCC |
| 18 –MSTLGVLLTICLLLFPLTALPLDGDQPADQSAERPAERTQDDIQQHPLYDPKRR-CCRYPCPDSCHGSCCYK ^3^ |
| 19 -----------------------EGDQPVNLPALRTQDFAP-ER--SPWFDPVKR-CCSRDCWVCIPCCPNGS ^2, 3^ |
| 20 ---------MLLLLLLLLPLAPGEGDEPVDLPALRTQDFAP-EH--SPWFDPVKR-CCSQDCLVCIPCCPNGSA |
| 21 -MSKLGVVLLVFLVLLALTSPLQNGNRFAGNQARKVGVQHRKNGLASALRRSSCGYLGQPCCVVPRRAYCHGDLECNDVTMCV |
| O1 superfamily: C-C-CC-C-C |
| 22 MKLTGVLIISVLLLTASQLITADYSRDKRQYLA------------MRLRDGLRN-----FRGVRDCGEQGEGCYTR-P----CCSGLTCVGTPVGGLCQP |
| 23 MKLTCALIVAMLLLTACQLTTTDDSRGRQKYPT------------ERLRAKMRNSK--LFKLTKRCDPPGDSCSRVYN---DCCSNY-CILRQSGPTC |
| 24 MKLTCMMIVAVLFLTAWTFVTADDSKNALENRG------GWGKPFPEARDEMKNPKASEL---RECSSYDEYCFPGIR---DCCSGF-CFIICI |
| 25 MKRTCALIVAVLFLTACQIIATNDSRGRQKYPT------------EKLRAKMRNSE--LFKLTKRCDPPGAGCSLWES---DCCSHF-CILRQSGPTC  26 MKLTCMMIVALLFLTAWTFVTAVDSKNELENRGGWGQAGGWGKLFLMARDEMKNSDVSKLDNRGRCVKDGDACVFPVVGSVFCCSGF-CVFVCI  27 MKLTCMMIVAVLFLTAL----ADDSRNGLENRN------------EQERNEK------EMRDRGRCRPGGVVCGFPKP-GPYCCSGW-CFFVC  28 --------------TASQLVTADYTRDKWQYPA------------ASLRGGMWN-----LRDTRACSQVGEACLPQKP----CCPGCLCNHIGGMCHH  29 --------------------------NGLENRN------------EQERNEN------EMRDRRDCQDSGVVCGFPKP-EPHCCSGW-CLFVCA ^2, 3^ |
| O2 superfamily: C-C-CC-C-C |
| 30 MEKLTILLLVAAVLMSTQALIQDGGEKRPKEKIKFLSKRKSVAESWWEGECSGWSNGCSWDSECCSGECIAGYCDFW |
| 31 MEKLAILLLAAAILMLTQALIQDGE-KHQRMKTHFLSKRKSVVESWWEGECSGWSVYCVNDWECCSGECGGSYCELW ^3^ |
| 32 MEKLTILLLVAAVLMSTQALIQDGGEKRQKEKIKFLSKRKSVAESWWEGECSGWSNGCYWDSECCSGECTQGYCDLW  O3 superfamily: C-C-CC-C-C  33 MSGLGTMVLTLLLLVFMVTSHQDGGEKQATQRNAINVGRRKSITRRTVDEECNEYCEDRDQNCCGKTNGEPRCAGICLG |
| T superfamily: CC-CC  34 MRCVPVFIILLLLSPSAPSVDAHPMTKDDVPQASLHDDAKRTLQVPWMKRGCCAMLTCCVGR ^2^ |
| V superfamily: C-C-CC-C-C-C-C |
| 35 MTPFILLLLFSLTIRCGDGKAIQEDRDPSAGLLWGDKNHDLSVKRRCFTCNGGECCGSCMCSWDKDDCICV |
| 36 MTPFILLLLFSLTIRCGDGKAIQEDRDPSAGLLTGDKNHNLSVKSGCGTCDGKTCCGRCECIWGNCRCRPWGK |
| 37 MTPFILLLLFSLTIRCGDGKAIQEDRDPSAGLLTGDKNHDLSVKRGCGTCDGKTCCGSCECPWGNCTCRKWGK |
| Y superfamily: C-C-CC-C-CC-C |
| 38 MQKATVLILAILLLLPLSTAQDDEGSQENAAERELARTTHCGGTGESCGRPEDPNCCGLQKCVSSKCCPTTDAC |
| Con-ikot-ikot: CC-C-C-C-C-CC-C-C-C-C  39 MAMNMSMTLIVFVMVFILATVIDSTLLQEPDLSRVKRTTRDCCIEATYQCLLNHHCQEHAHVTGCHWEAAGPCGVDPVEGCCYGYMYCMGMW  MEACNIDPVHNECKNEYCYTP  geneSuperfamily=Divergent M---L-LTVA: C-C-C-C-C-C  40 ------LVTVTLLLTLVMSIDSVPADETETGRVSLREDDR--FPCNSNQCACLPAEGSSTSYQCQSLDASTDDCFDNECVTQSEW  41 ------LATVALLLTLVMGIDSAPAGQTEAGRVSLREGLENTFPCNPGRCACLPASGTTTSYVCQSTQSSTADCMDNECVTEAEW  Unknown superfamily: C-C-C-C-C-C  42 ------------------ICRLEADVGPCSGTFPRWFYNSDMSKCQLFDYGGCRGNENRFDTEEECMELC |
| 43 -----------------DICRMPKVVGPCRAGITRYYYDTASAACRQFIYGGCQGNLNNFGSLEACQGKCAHH |
| 44 ------------------LCRLPAVPGPCRARQPRYFYNYKVGKCQRFNYGGCKGNTNRFLTLGECQTRC |
| 45 -------------------CNLPKIVGPCKAYMPSFFYNTGTGQCERFVYGGCGGNANRFETKQECEGQCQR |
| 46 ------------------VCRMPKDSGPCRASIPRWYYDANTRSCRQFVYGGCQGNGNNFESQQDCQDYC  47 VLLSVALFTQMATLRAEDICQQPLMAGRCQDVYERFFFNTSSGTCEAFIWGGCDGNANNFETFEACLAVC  48 ----LLLTAMVIVVATGNICRLERAIGPCRAAIRQYYYNWEARQCQTFIYGGCGGNDNRFETLEECEQAC |

^1^Signal regions are shown in red, mature regions are shown in green, and cysteine residues are marked in bold black. The sequences isolated and identified previously are highlighted in grey shading. ^2^Mature peptides in the sequences were aligned with previously recorded conotoxins in ConoServer. ^3^Mature peptides in the sequences were aligned with previously recorded conotoxins in GenBank.
