# Supplementary material for: High-Throughput Identification and Analysis of Novel Conotoxins from Three Vermivorous Cone Snails by Transcriptome Sequencing
Source: Mar Drugs. 2019 Mar 26;17(3):193. doi: 10.3390/md17030193 (PMC6471451; doi:10.3390/md17030193)
Supplement: Supplementary file 1 [file marinedrugs-17-00193-s001.zip › Supplementary Table 4-459494.docx]

**Supplementary Table S4.** The top 10 conotoxins with the highest FPKM values in the 3 transcriptome datasets.

| Serial no. | Superfamily | | | | RPKM value | | Total reads | | Gene ID |
| --- | --- | --- | --- | --- | --- | --- | --- | --- | --- |
| *C. caracteristicus* | | |  |  | |  | |  | |
| Ca-78 | O3 | | | | 425992.2 | | 248240 | | P02723-D1 |
| Ca-64 | O1 | | | | 382869.2 | | 108695 | | P00745-D8 |
| Ca-43 | M | | | | 340790.8 | | 175676 | | P04817-D5 |
| Ca-49 | O1 | | | | 331144 | | 180599 | | P00736-D1 |
| Ca-87 | T | | | | 271919.1 | | 140173 | | P02817-D1 |
| Ca-12 | B | | | | 261165.2 | | 103411 | | P04500-D3 |
| Ca-36 | L | | | | 220779.1 | | 103914 | | P00027-D1 |
| Ca-82 | O3 | | | | 180447.2 | | 60665 | | P00764-D4 |
| Ca-55 | O1 | | | | 180097.7 | | 104949 | | P01133-D1 |
| Ca-4 | A | | | | 175436.6 | | 81262 | | P00204-D1 |
| *C. generalis* | |  | |  | |  | |  | |
| Ge-5 | D | | | | 478573 | | 90086 | | P03588-D1 |
| Ge-33 | O2 | | | | 443511.7 | | 62839 | | P01115-D1 |
| Ge-34 | O2 | | | | 374137.5 | | 53767 | | P00698-D2 |
| Ge-16 | M | | | | 326621.6 | | 43633 | | P04876-D1 |
| Ge-40 | S | | | | 114047 | | 18698 | | P02821-D1 |
| Ge-22 | O1 | | | | 110041.3 | | 18264 | | P01111-D1 |
| Ge-41 | T | | | | 88309.9 | | 10546 | | P00655-D1 |
| Ge-31 | O2 | | | | 86311.8 | | 13452 | | P02758-D1 |
| Ge-1 | A | | | | 72318.1 | | 8929 | | P00123-D1 |
| Ge-23 | O1 | | | | 50633.8 | | 7584 | | P01182-D1 |
| *C. quercinus* | |  | |  | |  | |  | |
| Qu-5 | A | | | | 1128850 | | 230999 | | P00558-D1 |
| Qu-20 | M | | | | 879899.5 | | 274585 | | P04864-D1 |
| Qu-18 | M | | | | 818345 | | 288868 | | P01467-D1 |
| Qu-22 | O1 | | | | 274250.7 | | 109435 | | P01066-D1 |
| Qu-25 | O1 | | | | 229922.3 | | 94099 | | P01121-D2 |
| Qu-41 | Divergent M—L-LTVA | | | | 229648.3 | | 92812 | | P04047-D1 |
| Qu-4 | A | | | | 226257.3 | | 47457 | | P00551-D2 |
| Qu-17 | M | | | | 158874.2 | | 52830 | | P04845-D2 |
| Qu-12 | M | | | | 149633 | | 54350 | | P01083-D1 |
| Qu-32 | O2 | | | | 116437.4 | | 45271 | | P01115-D1 |
