# Supplementary material for: High-Throughput Identification and Analysis of Novel Conotoxins from Three Vermivorous Cone Snails by Transcriptome Sequencing
Source: Mar Drugs. 2019 Mar 26;17(3):193. doi: 10.3390/md17030193 (PMC6471451; doi:10.3390/md17030193)
Supplement: Supplementary file 1 [file marinedrugs-17-00193-s001.zip › Supplementary Table 5-459494.docx]

**Supplementary Table S5.** Conotoxin biosynthesis related proteins identified from the 3 venom duct transcriptomes.

| Homologous protein | Max E-value | Best score | Potential function |
| --- | --- | --- | --- |
| Protein disulfide isomerase | 0 | 976 | Disulfide bond formation |
| Tyrosylsulfotransferase | 1.00E-115 | 407 | Sulfation of tyrosine |
| Peptidyl-prolyl cis-trans isomerase B | 1.00E-109 | 386 | *Cis-trans* isomerization of proline |
| Peptidyl-prolyl cis-trans isomerase A | 7.00E-83 | 298 | *Cis-trans* isomerization of proline |
| Prolyl -hydroxylase | 1E-138 | 482 | Hydroxylation of Pro |
| Lysyl-hydroxylase | 6.00E-65 | 237 | Hydroxylation of Lys |
| Vitamin K-dependent γ-carboxylase | 0 | 1,540 | Carboxylation of glutamic acid |
| Peptidylglycine α-amidating monooxygenase | 0 | 1117 | Amidation of C-terminus |
| Glutaminyl-peptide cyclotransferase | 7.00E-24 | 101 | Cyclization of N-terminal Glu |
| Bromoperoxidase | 5.00E-11 | 61 | Bromination of tryptophan |
| Polypeptide Hex NAC  transferase | 1.00E-135 | 473 | O-glycosylation |
| Carboxypeptidase | 1.00E-152 | 531 | Proteolytic processing |
| Endoprotease | 1.00E-58 | 218 | Proteolytic processing |
| Ty3/Gyps yretrotransposon | 0 | 385 | Transposable element |
| Tc1-like elements | 5.00E-84 | 317 | Transposable element |
| XBP-1protein | 1.00E-44 | 183 | Peptide/Protein folding |
| 40S ribosomal protein | 1.00E-131 | 472 | Protein translation process |
| 60S ribosomal protein | 0 | 698 | Protein translation process |
| 60S acidic ribosomal protein P0 | 1.00E-127 | 456 | Protein translation process |
| 60S acidic ribosomal protein P1 | 7.00E-40 | 162 | Protein translation process |
| 60S acidic ribosomal protein P2 | 1.00E-37 | 156 | Protein translation process |
| aminoacyl tRNA synthetase | 0 | 874 | tRNA synthesis |
| pre-rRNA processing protein TSR1 | 1.00E-121 | 388 | Protein translation process |
| immunoglobulin binding protein | 2.00E-53 | 210 | Disulfide bond formation |
| 60kDa heat shock protein | 0 | 770 | Chaperone in protein folding and disulfide bond formation |
| 70kDa heat shock protein | 0 | 645 | Chaperone in protein folding and disulfide bond formation |
| 84 kDa heat shock protein | 0 | 1249 | Chaperone in protein folding and disulfide bond formation |
| 78kD glucose regulated protein | 0 | 1,116 | Chaperone in protein folding and disulfide bond formation |
| DNA J（Hsp40）protein | 1.00E-156 | 559 | Chaperone in protein folding |
| chaperonin | 0 | 857 | Co-chaperone in protein folding |
| calnexin | 0 | 766 | Co-chaperone in protein folding |
| calreticulin | 0 | 681 | Co-chaperone in protein folding |
| translocon Sec61 | 0 | 894 | Peptide binding and transport |
| translocon Sec14 | 1.00E-169 | 595 | Peptide binding and transport |
| translocon Sec31 | 0 | 1,,045 | Peptide binding and transport |
| translocon Sec24 | 0 | 926 | Peptide binding and transport |
| transmembrane protein TM9S3 | 0 | 960 | Transmembrane transport |
| transmembrane protein TM87A | 1.00E-122 | 438 | Transmembrane transport |
| Echotoxin B1 | 4.00E-50 | 203 | Pore forming enzyme |
| Echotoxin A | 6.00E-49 | 198 | Pore forming enzyme |
| Serine endopeptidase | 1.00E-91 | 338 | Antihemostatic role |
| Serine protease inhibitor | 1.00E-53 | 216 | hemostatic |
| hyaluronidase | 3.00E-58 | 231 | Venom diffusion factor |
| Metalloprotease | 1.00E-116 | 425 | Antihemostatic role |
| insulin | 1.00E-94 | 280 | hypoglycemic shock |
| C-type lectin | 1.00E-119 | 431 | Antihemostatic/myotoxic |
| galectin -2 | 1.00E-148 | 532 | Antihemostatic |
| Phospholipase A2 | 0 | 691 | neurotoxic |
| Phosphodiesterase | 0 | 669 | neurotoxic |
| nucleotidase | 0 | 943 | myotoxic |
| Cystein-rich secretory protein | 5.00E-27 | 129 | Cleavage of the mature conopeptides |
| ferritin | 5.00E-81 | 306 | Regulation of synthesis metabolism |
| Cytochrome P450 monooxygenase | 0 | 644 | Biotransformation of toxic exogenous compounds |
| Multi-copper oxidase | 1.00E-142 | 512 | Biotransformation of toxic exogenous compounds |
| superoxide dismutase | 2.00E-92 | 345 | cell protection against superoxide |
| inhibitor of apoptosis proteins | 0 | 1,104 | inhibition of cell apoptosis |
| Defensin | 3.00E-19 | 98 | Innate immunitu-related antimicrobial peptides |
| peptidoglycan recognition protein | 1.00E-52 | 212 | antimicrobial |
| ubiquitin | 2.00E-67 | 259 | Degradation of polypeptide |
| ubiquitin-conjugating  enzyme E2 | 1.00E-146 | 524 | Degradation of polypeptide |
| ubiquitin-protein ligase | 0 | 806 | Degradation of polypeptide |
| 26S proteasome | 0 | 1,334 | Degradation of polypeptide |
